# Supplementary material for: Pediatric Antibiotic Use Associated With Respiratory Syncytial Virus and Influenza in the United States, 2008–2018
Source: J Infect Dis. 2025 Jun 7;232(5):e811–23. doi: 10.1093/infdis/jiaf309 (PMC12614973; doi:10.1093/infdis/jiaf309)

SUPPORTING INFORMATION

Pediatric antibiotic use associated with respiratory syncytial virus and influenza in the United States, 2008-2018

Laura M King,[1] Katia J Bruxvoort,[2] Sara Y Tartof,[3] Joseph A Lewnard[1]
[1]University of California, Berkeley, School of Public Health, Berkeley, California, USA; [2]University of Alabama at Birmingham, Birmingham, Alabama, USA; [3]Kaiser Permanente Department of Research & Evaluation Southern California, Pasadena, California, USA

SUPPORTING INFORMATION

Table S1. Recommended antibiotic agents by condition

Table S2. Diagnosis categories and associated *International Classification of Diseases 9th* and *10th revision, Clinical Modification* codes

Table S3. Antibiotic class categorization

Table S4. Respiratory syncytial virus-, influenza-, and bronchiolitis-associated visits by age category.

Table S5. All-cause antibiotic prescription incidence by condition and age group.

Table S6. Sensitivity analysis: model-estimated proportion of outpatient antibiotic prescriptions associated with respiratory syncytial virus (RSV) infection in analyses using bronchiolitis visits in children 0-2 years as a proxy for RSV activity.

Table S7. Sensitivity analysis: model-estimated proportion of outpatient antibiotic prescriptions associated with influenza virus infection in analyses using bronchiolitis visits in children 0-2 years as respiratory syncytial virus incidence proxy.

Table S8. Incidence of respiratory syncytial virus-, influenza-, and bronchiolitis-associated visits by census division.

Table S9. Model-estimated proportion of outpatient antibiotic prescriptions associated with respiratory syncytial virus and influenza virus infection, by census division.

Table S10: Sensitivity analysis – model-estimated proportion of outpatient antibiotic prescriptions associated with respiratory syncytial virus and influenza infection based on a linear model.

Table S11. Sensitivity analysis – model-estimated proportion of outpatient antibiotic prescriptions associated with RSV and influenza infection, when antibiotic definition is expanded to all antibiotics.

Table S12. Sensitivity analysis — incidence rates and model-estimated proportion of outpatient antibiotic prescriptions associated with respiratory syncytial virus and influenza virus infection in analyses applying broader inclusion criteria for prescription plan utilization.

Table S13. Incidence and proportion of outpatient antibiotic prescriptions associated with respiratory syncytial virus and influenza infection linked to diagnoses for which antibiotics are not indicated, without exclusion of prescriptions linked to other conditions, among children aged 0-17 years, 2008-2018.

Figure S1. Predicted and observed antibiotic prescription counts

Table S1. Recommended antibiotic agents[a] by condition.

Condition	Source	Agents[a]
Acute otitis media	Lieberthal et al. 2013	Amoxicillin-clavulanate, amoxicillin, cefdinir, cefuroxime, cefpodoxime, ceftriaxone, clindamycin, 3rd generation cephalosporin (cefixime, ceftaxidime, cefotaxime, ceftizoxime, cefpodoxime, ceftriaxone, cefoperazone, ceftibuten, cefdinir, cefditoren)
Sinusitis	Wald et al. 2013	Amoxicillin-clavulanate, amoxicillin, clindamycin, cefixime, linezolid, levofloxacin
Pharyngitis	Shulman et al. 2012	Penicillin V, amoxicillin, penicillin G, cephalexin, cefadroxil, clindamycin, azithromycin, clarithromycin
Pneumonia[b]	Bradley et al. 2011	Amoxicillin, amoxicillin-clavulanate, ampicillin, azithromycin, cefazolin, cefdinir, cefixime, cefotaxime, cefpodoxime, cefprozil, ceftibuten, ceftriaxone, cefuroxime, cephalexin, =floxacin, clarithromycin, clindamycin, doxycycline, erythromycin, levofloxacin, linezolid, moxifloxacin, penicillin, semisynthetic penicillin, vancomycin

[a] Includes both first-line and alternative agents. Some agents listed recommended in combination. We included all recommended agents in our analysis.

[b] Recommended agents varies by pathogen.

Table S2. Diagnosis categories and associated *International Classification of Diseases 9th* and *10th revision, Clinical Modification* codes.

Diagnosis category	ICD-9-CM codes	ICD-10-CM codes
RSV[a,b]	079.6, 466.11, 480.1	B97.4, J12.1, J20.5, J21.0
Influenza[b,c]	487*, 488*	J09*-J11*,
Acute otitis media[b]	382*, 384.0*, 384.1	H66*, H67*, H73-H73.23
Acute exacerbation of COPD[b]	491.21, 491.22, 493.21, 493.22, 494.1	J44, J44.0-J44.1
Allergy[b]	477*, 995.3	J30*, T78.4, T78.40*, T78.49*
Asthma[b]	493.0*, 493.1*, 493.20, 493.8*, 493.9*,	J45*
Bronchiolitis[a,b]	466.1, 466.19	J21, J21.1-J21.9
Bronchitis[a,b]	466.0, 490	J20-J20.4, J20.6-J20.9, J40
Pharyngitis[b]	034.0, 462, 463	J02*, J03*
Sinusitis[b]	461*, 473*	J01*, J32*
Pneumonia[a,b,d]	055.1, 073.0, 480.0, 480.2-486, 507.0, 516.36, 517.1, 770.0, 997.31, 997.32	A48.1, B01.2, B05.2, B06.81, J12, J12.0, J12.2-J18.9, J69, J69.0, J84.116, J95.851, O29.01*, O74.0, O89.01, P23*
Non-suppurative otitis media[b]	381*	H65*, H68*, H69*
Viral URI[b]	460, 464.0-464.21, 464.4-464.59, 786.2	J00, J04,*J05.0, J06*, R05
Urinary tract infection[e]	590.00- 590.11, 590.8-590.9, 595.0-595.2, 595.4, 595.81, 595.89, 595.9, 596.8, 596.81, 599.0, 646.5*, 771.82,	N10, N11- N11.1, N13.6, N30-N30.21, N30.8-N30.91, N39.0, N99.511, N99.521, N99.531, O03.38, O03.88, O04.88, O07.38, O08.83, O23-O23.13, O23.3- O23.43, O86.2*, P39.3
Other bacterial infections[e]	003.1, 003.21-003.24, 006.3-006.8, 010-033.9, 034.1, 036-041.9, 073.7-073.9, 076-077.0, 077.98, 078.2, 078.3, 078.82, 078.88, 079.88, 079.9, 079.98, 080-084.9, 087-102.9, 104*, 130-131.9, 136.3-137.4, 139.1, 139.8, 254.1, 320-321.1, 321.8-322.9, 323.1-324.9, 326, 349.82, 357.0, 360.00, 360.02, 360.04, 376.02-376.04, 376.12, 379.45, 379.60-379.63, 380.14, 383.0-383.22, 383.81, 383.9, 390-393, 394.1, 395*, 397.1-398.99, 415.12, 420-421.9, 422.92, 424.9*, 440.24, 449, 464.30, 464.31, 475, 478.22, 478.24, 511.1, 513.0, 513.1, 517.3, 519.01, 519.2, 530.86, 536.41, 539.01, 539.81, 540-542, 550.0*, 551*, 564.7, 566-567.81, 567.89, 567.9, 569.5, 569.61, 569.83, 572.0, 572.1, 573.2, 575.4, 576.1, 576.3, 590.2, 597.0, 597.80, 597.89, 598.00, 598.01, 601.0-601.4, 601.9, 603.1, 604*, 607.2, 614.0-614.2, 614.5-616.11, 616.3-616.51, 616.89, 616.9, 634.00-634.02, 635.00-635.02, 636.00-636.02, 637.00-637.02, 638.0, 639.0, 646.60-646.64, 647.00-647.44, 647.80-647.94, 658.4*, 659.3*, 670*, 673.3*, 675.1*, 695.81, 711.00-711.19, 711.9*, 728.0, 728.86, 730.00-730.29, 730.80-730.99, 760.2, 770.12, 771.2-771.4, 771.81, 771.83, 771.89, 777.5*, 777.6, 785.4, 785.52, 788.7, 790.7, 795.3*, 958.3, 995.91, 995.92, 996.6*, 997.62, 998.02, 998.51, 998.59, 999, 999.31, 999.32, 999.34, 999.39, V01.0, V01.1, V01.6, V01.81, V01.83-V01.89, V01.9, V02.8, V09*, V73.88, V73.98	A01, A01.0, A01.01-A01.05, A02, A02.1, A02.2, A02.21-A02.29, A06, A06.4-A06.82, A15*-A44*, A48, A48.0, A48.2-A59.9, A63, A63.8, A64*-A66*, A68*-A79*, B47, B47.1-B47.9, B50*-B54*, B58-B60.0, B60.2, B90*, B92-B94.0, B94.8-B96.89, B99*, D57.01, D57.211, D57.411, D57.811, D73.3, E08.52, E09.52, E10.52, E11.52, E13.52, E32.1, G00-G03.1, G03.8-G04.02, G04.2-G07, G92, H05, H05.0, H05.02-H05.049, H05.1, H05.12*, H44, H44.0*, H57.01, H59.4-H59.43, H60.2*, H70*, H75*, I00-I09.9, I30*, I32-I33.9, I38, I39, I40.0, I70.26*, I70.36*, I70.46*, I70.56*, I70.66*, I70.76*, I73.01, I76, I96, J05, J05.1*, J36, J39.0, J39.1, J47-J47.1, J65, J80, J85-J86.9, J98.5, J98.51, K35-K37, K40.1*, K40.4*, K41.1*, K41.4*, K42.1, K43.1, K43.4, K43.7, K44.1, K45.1, K46.1, K50.014, K50.114, K50.814, K50.914, K51.014, K51.214, K51.314, K51.414, K51.514, K51.814, K51.914, K55.3*, K57.0*, K57.2*, K57.4*, K57.8*, K59.3*, K61*, K63.0, K63.1, K65-K65.3, K65.8, K65.9, K68-K68.19, K75.0, K80.3*, K82.2, K82.A*, K83.0*, K83.2, K85.02, K85.12, K85.22, K85.32, K85.82, K85.92, K90.81, K94.02, K94.12, K94.22, K94.32, K95.01, K95.81, L00, M00-M01.X9, M02.3*, M46.2-M46.39, M46.5*, M60.0*, M65.0-M65.19, M71.0 -M71.19, M72.6, M86*, N15.1, N34-N34.2, N41-N41.3, N43.1, N45,*, N48.2, N48.21, N49.3, N70-N73, N73.3-N74, N75.1, N76-N76.6, N77*, N98.0, O03.0, O03.37, O03.5, O03.87, O04.5, O04.87, O07.0, O07.37, O08.0, O08.82, O23.2*, O23.5*. O23.9*, O41.1*, O75.3, O85-O86.19, O86.8*, O88.3*, O91.1*, O98-O98.33, O98.8-O98.93, O99.8, O99.82-O99.835, P00.2, P02.7*, P24.0, P24.01, P36-P37.2, P37.8-P39, P39.2, P39.8, P39.9, P58.2, P77*, P78.0, R36, R36.0, R36.9, R65, R65.2-R65.21, R78.81, T80.2-T80.211S, T80.218-T80.29XS, T81.12*, T81.4*, T82.6-T82.7XXS, T83.5-T83.6XXS, T84.5-T84.7XXS, T85, T85.7*, T86.03, T86.13, T86.23, T86.33, T86.43, T86.812, T86.832, T86.852, T86.892, T86.93, T87.4*, Z11.3, Z20, Z20.1, Z20.2, Z20.8, Z20.81*, Z20.89, Z20.9, Z71.84

Diagnosis category	ICD-9-CM codes	ICD-10-CM codes
Gastrointestinal infections	001.0-003.0, 003.20, 003.29-006.2, 006.9-009.3, 522.0, 522.5, 522.7, 526.5, 527.2-, 527.3, 533*, 562.01, 562.03, 562.11, 562.13, 569.7*, 569.87, 577.0, 579.1, 579.2, 779.32, 787.04, 787.6*, 787.91, 789.1, 789.2	A00, A00.0-A00.1, A00.9, A01.00, A01.09, A01.1-A01.4, A02.0, A02.20, A02.8-A05.9, A06.0-A06.3, A06.89-A09, K04-K04.1, K04.6-K04.7, K11, K11.2-K11.3, K11.8, K11.9, K27*, K51.5-K51.513, K51.518-K51.519, K57, K57.1, K57.12, K57.13, K57.3, K57.32, K57.33, K57.5, K57.52, K57.53, K57.9, K57.92, K57.93, K67, K85-K85.01, K85.1-K85.11, K85.2-K85.21, K85.3-K85.31, K85.8-K85.81, K85.9-K85.91, K90.1-K90.2, K91.85-K91.858, M27.3, M27.51, P78.1, P78.82, R11.12-R11.14, R15-R16.2, R19.7, Z20.0-Z20.09
Skin and soft tissue infections	035, 103*, 110-111.9, 360.01, 360.03, 360.11-360.13, 360.19, 363.00-363.21, 364.00-364.03, 364.05-364.21, 364,3, 370.00-370.22, 370.31, 370.40-370.52, 370.55, 370.59, 370.8, 370.9, 372.00-372.04, 372.10-372.12, 372.15-372.21, 372.30-372.33, 372.39, 373.00-373.2, 373.6, 375.01, 375.02, 375.30-375.42, 376.01, 376.13, 380.00-380.13, 380.15, 380.16, 380.22, 380.23, 457.2, 478.21, 478.71, 528.0-528.3, 611.0, 611.5, 614.3, 614.4, 675.0*, 675.20-675.94, 680.0-686.09, 686.8, 686.9, 694.1, 694.3, 695.3, 705.82, 705.83, 706.0, 706.1, 723.6, 729.30, 729.39, 771.5, 771.6, 910.1, 910.3, 910.5, 910.7, 910.9, 911.1, 911.3, 911.5, 911.7, 911.9, 912.1, 912.3, 912.5, 912.7, 912.9, 913.1, 913.3, 913.5, 913.7, 913.9, 914.1, 914.3, 914.5, 914.7, 914.9, 915.1, 915.3, 915.5, 915.7, 915.9, 916.1, 916.3, 916.5, 916.7, 916.9, 917.1, 917.3, 917.5, 917.7, 917.9, 919.1, 919.3, 919.5, 919.7, 919.9, 999.33, E90.60-E90.63, E90.65, E92.83, E96.87	A46, A67*, B35*-B36*, H00-H01.02B, H04-H04.029, H04.3-H04.429, H05.01*, H10-H10.029, H10.2, H10.22-H10.409, H10.42-H10.439, H10.5-H10.509, H10.52*, H10.8, H10.82-H10.9, H16-H16.119, H16.14-H16.209, H16.25*, H16.29-H16.329, H16.39*, H16.8, H16.9, H20-H20.039, H20.05-H20.13, H20.8-H20.819, H20.9, H21.33*, H33.12*, H44.1*, H60-H60.13, H60.3*, H60.6-H61.039, H62-H62.43, H94-H94.03, I89.1, J34.0, J95.02, K12-K12.39, L01-L08.9, L66.3, L70*, L71, L71.8, L71.9, L73*, L88, M79.3, N48.22, N61*, N73.0-N73.2, O91-O91.03, O91.2-O91.23, P39.0, P39.1, P39.4, S00.07, S00.07XA, S00.27, S00.271, S00.271A, S00.272, S00.72A, S00.279, S00.279A, S00.37, S00.37XA, S00.47, S00.471, S00.471A, S00.472, S00.472A, S00.479, S00.479A, S00.57, S00.571, S00.571A, S00.572, S00.572A, S00.87, S00.87XA, S00.97, S00.97XA, S01.05, S01.05XA, S01.15, S01.151, S01.151A, S01.152, S01.152A, S01.159, S01.159A, S01.25, S01.25XA, S01.35, S01.351, S01.351A, S01.352, S01.352A, S01.359, S01.359A, S01.45, S01.451, S01.451A, S01.452, S01.452A, S01.459, S01.459A, S01.55, S01.551, S01.551A, S01.552, S01.552A, S01.85, S01.85XA, S01.95, S01.95XA, S10.17, S10.17XA, S10.87, S10.87XA, S10.97, S10.97XA, S11.015, S11.015A, S11.025, S11.025A, S11.035, S11.035A, S11.15, S11.15XA, S11.25, S11.25XA, S11.85, S11.95, S11.95XA, S20.17, S20.171, S20.171A, S20.172, S20.172A, S20.179, S20.179A, S20.37, S20.371, S20.371A, S20.372, S20.372A, S20.379, S20.379A, S20.47, S20.471, S20.471A, S20.472, S20.472A, S20.479, S20.479A, S20.97, S20.97XA, S21.05, S21.051, S21.051A, S21.052, S21.052A, S21.059, S21.059A, S21.15, S21.151, S21.151A, S21.152, S21.152A, S21.159, S21.159A, S21.25, S21.251, S21.251A, S21.252, S21.252A, S21.259, S21.259A, S21.35, S21.351, S21.351A, S21.352, S21.352A, S21.359, S21.359A, S21.45, S21.451, S21.451A, S21.452, S21.452A, S21.459, S21.459A, S21.95, S21.95XA, S30.87, S30.870, S30.870A, S30.871, S30.871A, S30.872, S30.872A, S30.873, S30.873A, S30.874, S30.874A, S30.875, S30.875A, S30.876, S30.876A, S30.877, S30.877A, S31.05, S31.050, S31.050A, S31.051, S31.051A, S31.15, S31.150, S31.150A, S31.151, S31.151A, S31.152, S31.152A, S31.153, S31.153A, S31.154, S31.154A, S31.155, S31.155A, S31.159, S31.159A, S31.25, S31.25XA, S31.35, S31.35XA, S31.45, S31.45XA, S31.55, S31.551, S31.551A, S31.552, S31.552A, S31.65, S31.650, S31.650A, S31.651, S31.651A, S31.652, S31.652A, S31.653, S31.653A, S31.654, S31.654A, S31.655,

Diagnosis category	ICD-9-CM codes	ICD-10-CM codes
		S31.655A, S31.659, S31.659A, S31.805, S31.805A, S31.815, S31.815A, S31.825, S31.825A, S31.835, S31.835A, S40.27, S40.271, S40.271A, S40.272, S40.272A, S40.279, S40.279A, S40.87, S40.871, S40.871A, S40.872, S40.872A, S40.879, S40.879A, S41.05, S41.051, S41.051A, S41.052, S41.052A, S41.059, S41.059A, S41.15, S41.151, S41.151A, S41.152, S41.152A, S41.159, S41.159A, S50.37, S50.371, S50.371A, S50.372, S50.372A, S50.379, S50.379A, S50.87, S50.871, S50.871A, S50.872, S50.872A, S50.879, S50.879A, S51.05, S51.051, S51.051A, S51.052, S51.052A, S51.059, S51.059A, S51.85, S51.851, S51.851A, S51.852, S51.852A, S51.859, S51.859A, S60.37, S60.371, S60.371A, S60.372, S60.372A, S60.379, S60.379A, S60.47, S60.470, S60.470A, S60.471, S60.471A, S60.472, S60.472A, S60.473, S60.473A, S60.474, S60.474A, S60.475, S60.475A, S60.476, S60.476A, S60.477, S60.477A, S60.478, S60.478A, S60.479, S60.479A, S60.57, S60.571, S60.571A, S60.572, S60.572A, S60.579, S60.579A, S60.87, S60.871, S60.871A, S60.872, S60.872A, S60.879, S60.879A, S61.05, S61.051, S61.051A, S61.052, S61.052A, S61.059, S61.059A, S61.15, S61.151, S61.151A, S61.152, S61.152A, S61.159, S61.159A, S61.25, S61.250, S61.250A, S61.251, S61.251A, S61.252, S61.252A, S61.253, S61.253A, S61.254, S61.254A, S61.255, S61.255A, S61.256, S61.256A, S61.257, S61.257A, S61.258, S61.258A, S61.259, S61.259A, S61.35, S61.350, S61.350A, S61.351, S61.351A, S61.352, S61.352A, S61.353, S61.353A, S61.354, S61.354A, S61.355, S61.355A, S61.356, S61.356A, S61.357, S61.357A, S61.358, S61.358A, S61.359, S61.359A, S61.45, S61.451, S61.451A, S61.452, S61.452A, S61.459, S61.459A, S61.55, S61.551, S61.551A, S61.552, S61.552A, S61.559, S61.559A, S70.27, S70.271, S70.271A, S70.272, S70.272A, S70.279, S70.279A, S70.37, S70.371, S70.371A, S70.372, S70.372A, S70.379, S70.379A, S71.05, S71.051, S71.051A, S71.052, S71.052A, S71.059, S71.059A, S71.15, S71.151, S71.151A, S71.152, S71.152A, S71.159, S71.159A, S80.27, S80.271, S80.271A, S80.272, S80.272A, S80.279, S80.279A, S80.87, S80.871, S80.871A, S80.872, S80.872A, S80.879, S80.879A, S81.05, S81.051, S81.051A, S81.052, S81.052A, S81.059, S81.059A, S81.85, S81.851, S81.851A, S81.852, S81.852A, S81.859, S81.859A, S90.47, S90.471, S90.471A, S90.472, S90.472A, S90.473, S90.473A, S90.474, S90.474A, S90.475, S90.475A, S90.476, S90.476A, S90.57, S90.571, S90.571A, S90.572, S90.572A, S90.579, S90.579A, S90.87, S90.871, S90.871A, S90.872, S90.872A, S90.879, S90.879A, S91.05, S91.051, S91.051A, S91.052, S91.052A, S91.059, S91.059A, S91.15, S91.151, S91.151A, S91.152, S91.152A, S91.153, S91.153A, S91.154, S91.154A, S91.155, S91.155A, S91.156, S91.156A, S91.159, S91.159A, S91.25, S91.251, S91.251A, S91.252, S91.252A, S91.253, S91.253A, S91.254, S91.254A, S91.255, S91.255A, S91.256, S91.256A, S91.259, S91.259A, S91.35, S91.351,

Diagnosis category	ICD-9-CM codes	ICD-10-CM codes
		S91.351A, S91.352, S91.352A, S91.359, S91.359A, T80.212*, T86.822, T86.842, W50, W50.3, W50.3XXA, W53.01, W53.01XA, W53.11, W53.11XA, W53.21, W53.21XA, W53.81, W53.81XA, W54.0, W54.0XXA, W55.01, W55.01XA, W55.11, W55.11XA, W55.21, W55.21XA, W55.31, W55.31XA, W55.41, W55.41XA, W55.51, W55.51XA, W55.81, W55.81XA, W56.01, W56.01XA, W56.11, W56.11XA, W56.21, W56.21XA, W56.31, W56.31XA, W56.41, W56.41XA, W56.51, W56.51XA, W56.81, W56.81XA, W58.01, W58.01XA, W58.11, W58.11XA, W59.01, W59.01XA, W59.11, W59.11XA, W59.21, W59.21XA, W59.81, W59.81XA, W61.01, W61.01XA, W61.11, W61.11XA, W61.2, W61.21, W61.21XA, W61.51, W615.1XA, W61.61, W61.61XA, W61.91, W61.91XA, Y04.1, Y04.1XXA

Abbreviations: ICD-9-CM -- *International Classification of Diseases 9th revision, Clinical Modification;* ICD-10-CM -- *International Classification of Diseases 10th revision, Clinical Modification;* RSV – respiratory syncytial virus; COPD – chronic obstructive pulmonary disorder.

* Includes all child codes beginning with the numeric sequence.

[a] Categorized as lower respiratory tract infection (LRTI).

[b] Categorized as acute respiratory infection (ARI).

[c] Includes acute otitis media and pneumonia due to influenza.

[d] Includes viral pneumonia except pneumonia related to influenza, which is captured under influenza.

[e] Used to exclude antibiotic prescriptions from being attributed to ARI-related diagnoses when ARI and UTI or ARI and other bacterial infections are both present for a single visit.

Table S3. Antibiotic class categorization.

Class	Antibiotic agent[a]
Penicillin	Amoxicillin, ampicillin, penicillin G, penicillin V, carbenicillin, dicloxacillin
Extended-spectrum beta-lactam	Amoxicillin-clavulanate, am*picillin-sulbactam*
Macrolide	Azithromycin, clarithromycin, erythromycin, *erythromycin-sulfisoxazole*
Cephalosporin	Cefdinir, cefuroxime, cefpodoxime, ceftriaxone, cefixime, cefotaxime, cefditoren, ceftibuten, cephalexin, cefadroxil, cefazolin, cefprozil, *cefaclor, cefepime, ceftazidime*
Tetracycline	Doxycycline, *minocycline, tetracycline, demecylocycline*
Fluoroquinolone	Levofloxacin, moxifloxacin, ciprofloxacin, *delafloxacin, gatifloxacin, gemifloxacin, norfloxacin, oflaxacin, besifloxacin*
Other	Clindamycin, linezolid, vancomycin, *amikacin, chloramphenicol, colistimethate, colistin, fosfomycin, furazolidone, gentamycin, lincomycin, loracarbef, meropenem, methenamine, nitrofurantoin, rifaximin, secnidazole, sulfadiazine, sulfamethoxazole, sulfamethoxazole-trimethoprim, sulfanilamid, sulfisoxazole, tedizolid, telithromycin, trimethoprim,*

[a] Includes agents with >1 dispensing event in the Clinformatics database. Both agents classified as respiratory and non-respiratory included. Agents not categorized as respiratory (**Table S1**) are italicized. Only agents categorized as respiratory included in primary analyses, all agents included in sensitivity analysis.

Table S4. Respiratory syncytial virus-, influenza-, and bronchiolitis-linked visits by age category.

Age category	Visit[a] incidence per 1000 person-years (95% CI)[b]		
	RSV	*Influenza*	*Bronchiolitis*
0-2 years	207 (180, 236)	111 (91, 132)	527 (483, 573)
3-5 years	24 (15, 34)	139 (116, 162)	63 (48, 79)
6-9 years	6 (2, 11)	151 (128, 176)	15 (8, 24)
10-13 years	3 (0, 8)	124 (103, 146)	8 (3, 14)
14-17 years	3 (0, 6)	83 (66, 102)	5 (1, 10)
Total	53 (39, 68)	121 (100, 143)	101 (82, 121)

Abbreviations: CI – confidence interval; RSV – respiratory syncytial virus.

[a] All visits with any diagnostic code for RSV, influenza, or bronchiolitis as detailed in **Table S2.** We additionally included visits with ICD-9-CM code 466.11 or ICD-10-CM code J21.0 (bronchiolitis due to RSV) in the definition of bronchiolitis visits. Visits with diagnosis codes for multiple syndromes were included in all applicable measures. Visits with diagnosis codes for both influenza and RSV infections accounted for 0.4% of all visits for either syndrome.

[b] Obtained by fitting number of antibiotic prescriptions divided by person-years to a Poisson distribution, sampling 100,000 random draws, and obtaining the median, 2.5th and 97.5th percentile values.

Table S5. All-cause antibiotic prescription incidence by condition and age group.

Linked diagnosis[b]		Antibiotic prescription incidence per 1000 person-years (95% CI)[a]					
		All children	*0-2 years*	*3-5 years*	*6-9 years*	*10-13 years*	*14-17 years*
Any[c]		1,161 (1,096, 1,228)	1,834 (1,752, 1,917)	1,469 (1,396, 1,544)	1,129 (1,065, 1,195)	846 (790, 902)	897 (841, 956)
Respiratory syndromes							
	Any ARI[d,e]	775 (723, 830)	1,394 (1,323, 1,467)	1,081 (1,018, 1,145)	800 (747, 856)	554 (509, 600)	412 (373, 452)
	Any LRTI[e,f]	99 (81, 119)	149 (126, 173)	144 (121, 168)	101 (83, 122)	76 (60, 93)	62 (48, 78)
Specific condition							
	Pneumonia[e,g]	29 (20, 41)	41 (29, 54)	50 (37, 65)	34 (24, 47)	21 (13, 31)	12 (6, 19)
	AOM[e]	275 (244, 308)	864 (808, 922)	446 (406, 488)	210 (183, 239)	103 (84, 123)	55 (41, 70)
	Sinusitis[e]	147 (124, 171)	153 (130, 178)	170 (145, 196)	136 (114, 159)	140 (118, 164)	145 (123, 170)
	Pharyngitis[e]	229 (201, 260)	140 (117, 163)	275 (243, 308)	336 (301, 372)	233 (204, 263)	151 (128, 176)
	Bronchiolitis[h,i]	4 (1, 8)	17 (9, 25)	5 (1, 10)	2 (0, 5)	1 (0, 3)	0 (0, 2)
	Bronchitis[i,j]	42 (30, 55)	41 (29, 54)	60 (45, 75)	45 (33, 59)	37 (26, 49)	32 (22, 44)
	Nonsupp. OM[i]	32 (21, 43)	97 (79, 117)	52 (38, 66)	24 (15, 35)	12 (6, 19)	7 (2, 12)
	Allergy[i]	11 (5, 18)	8 (3, 14)	13 (7, 21)	13 (7, 21)	11 (5, 18)	9 (4, 15)
	Asthma[i]	16 (9, 25)	14 (7, 22)	22 (13, 31)	20 (12, 29)	16 (9, 24)	11 (5, 18)
	Viral URI (excl. RSV and influenza)[i]	75 (59, 93)	129 (108, 152)	109 (90, 130)	72 (56, 89)	54 (40, 69)	45 (33, 59)
	Any RSV diagnosis[i]	1 (0, 4)	6 (2, 11)	1 (0, 4)	0 (0, 2)	0 (0, 1)	0 (0, 1)
	Any influenza diagnosis[i,k]	4 (1, 9)	3 (0, 7)	5 (1, 10)	5 (2, 11)	4 (1, 9)	3 (0, 7)

Abbreviations: CI – confidence interval; ARI – acute respiratory infection; LRTI – lower respiratory tract infection; AOM – acute otitis media; Non-supp. OM – nonsuppurative otitis media; URI – upper respiratory infection; RSV – respiratory syncytial virus.

[a] Obtained by fitting number of antibiotic prescriptions divided by person-years to a Poisson distribution and sampling 100,000 random draws.

[b] Based on ICD-9-CM and ICD-10-CM codes (**Table S3**) assigned at the most recent outpatient visit within a 4-day window. More than one diagnosis can be associated with an antibiotic prescription.

[c] Includes antibiotic prescriptions for any diagnosis.

[d] Includes diagnoses of pneumonia, AOM, sinusitis, pharyngitis, bronchiolitis, bronchitis, non-suppurative otitis media, viral upper respiratory infection, RSV, influenza, and acute exacerbation of chronic bronchitis/obstructive pulmonary disorder (accounts for <0.04% of antibiotic prescriptions).

[e] Antibiotic prescriptions with concurrent diagnoses of urinary tract infection and other bacterial infections excluded.

[f] Includes diagnoses of bronchitis, bronchiolitis, and pneumonia excluding diagnosis codes for these conditions due to RSV or influenza.

[g] Excludes pneumonia due to influenza and RSV but includes other viral pneumonia. Among pneumonia prescriptions, 2.4% had a viral pneumonia diagnosis with no other pneumonia diagnosis.

[h] Excludes diagnosis codes for bronchiolitis due to RSV.

[i] Antibiotic prescriptions with concurrent diagnoses of urinary tract infection, other bacterial infections, pneumonia, AOM, sinusitis, pharyngitis, gastrointestinal infections, and skin and soft tissue infections excluded.

[j] Excludes diagnosis codes for bronchitis due to RSV.

[k] Includes all diagnoses of influenza, including influenza and pneumonia and influenza and AOM.

Table S6. Sensitivity analysis: model-estimated proportion of outpatient antibiotic prescriptions associated with respiratory syncytial virus (RSV) infection in analyses using bronchiolitis visits in children 0-2 years as a proxy for RSV activity.[a]

Linked diagnosis[b]		Percent of antibiotic prescriptions associated with RSV infection (95% CI)					
		All children	*0-2 years*	*3-5 years*	*6-9 years*	*10-13 years*	*14-17 years*
Any[c]		8.0 (6.8, 9.3)	11.2 (8.0, 14.4)	10.0 (6.7, 13.4)	6.9 (3.6, 10.2)	6.4 (4.0, 8.7)	4.3 (2.8, 5.8)
Respiratory syndromes							
	Any ARI[d,e]	9.2 (7.5, 10.9)	11.5 (7.9, 15.1)	11.4 (7.4, 15.4)	7.7 (3.4, 11.9)	7.7 (3.9, 11.5)	7.3 (4.3, 10.4)
	Any LRTI[e,f]	17.0 (15.2, 18.8)	32.2 (29.1, 35.2)	21.0 (17.0, 24.9)	12.0 (7.3, 16.4)	8.3 (3.6, 12.9)	7.7 (3.4, 11.8)
Specific conditions							
	Pneumonia[e,g]	15.4 (13.6, 17.2)	25.7 (22.5, 28.7)	23.0 (19.3, 26.5)	11.9 (7.6, 15.9)	6.6 (1.9, 11.1)	7.5 (2.8, 12.0)
	AOM[e]	12.3 (10.7, 14.0)	12.3 (8.6, 16.0)	16.0 (12.0, 19.8)	14.0 (9.9, 17.8)	10.3 (6.6, 13.9)	8.0 (4.7, 11.3)
	Sinusitis[e]	6.3 (4.7, 8.0)	3.9 (0.1, 7.6)	6.2 (2.2, 10.2)	5.8 (1.6, 9.9)	7.9 (4.1, 11.6)	8.5 (5.3, 11.6)
	Pharyngitis[e]	4.8 (2.9, 6.7)	4.3 (0.5, 7.9)	3.6 (-0.8, 7.9)	3.2 (-1.8, 7.9)	6.2 (1.6, 10.7)	7.0 (3.4, 10.5)
	Bronchiolitis[h,i]	26.7 (24.2, 29.1)	40.3 (37.1, 43.4)	29.8 (24.8, 34.6)	14.0 (5.8, 21.5)	8.6 (-2.3, 18.3)	4.2 (-8.4, 15.3)
	Bronchitis[i,j]	11.3 (9.0, 13.6)	15.5 (11.1, 19.7)	15.7 (10.9, 20.4)	10.3 (4.7, 15.6)	8.5 (2.7, 14.0)	6.4 (0.9, 11.6)
	Non-suppurative otitis media[i]	10.5 (8.8, 12.2)	11.1 (7.5, 14.6)	13.3 (9.4, 17.0)	11.4 (7.5, 15.2)	8.6 (4.9, 12.3)	6.6 (2.6, 10.4)
	Allergy[i]	5.5 (3.8, 7.3)	2.3 (-2.5, 7.0)	7.6 (3.8, 11.4)	4.2 (0.2, 8.0)	6.7 (3.0, 10.3)	4.2 (0.7, 7.6)
	Asthma[i]	9.6 (7.8, 11.5)	15.5 (11.7, 19.2)	11.0 (6.8, 15.0)	9.0 (4.6, 13.2)	7.7 (3.3, 12.0)	5.1 (0.9, 9.2)
	Viral URI[i] (excl. RSV and influenza)	11.0 (9.1, 12.8)	13.1 (9.4, 16.8)	13.5 (9.3, 17.6)	9.4 (4.6, 13.9)	9.9 (5.3, 14.4)	8.8 (4.6, 12.9)
	Any RSV diagnosis[i]	51.9 (49.6, 54.2)	63.3 (60.7, 65.8)	55.3 (50.7, 59.5)	26.0 (14.8, 35.3)	2.4 (-20.3, 20.2)	-0.5 (-26.2, 18.9)
	Any influenza diagnosis[i,k]	25.3 (21.9, 28.6)	23.1 (14.3, 31.0)	29.0 (21.2, 36.1)	26.2 (18.0, 33.4)	20.0 (12.8, 26.8)	26.1 (18.4, 33.2)

Abbreviations: CI – confidence interval; RSV – respiratory syncytial virus; ARI – acute respiratory infection; LRTI – lower respiratory tract infection; AOM – acute otitis media; Non-supp. OM – nonsuppurative otitis media; URI – upper respiratory infection.

[a] Primary analysis used the percent of samples positive for RSV measured from laboratory data from NREVSS lagged one week behind antibiotic prescriptions as the RSV transmission proxy.

[b] Based on ICD-9-CM and ICD-10-CM codes (**Table S3**) assigned at the most recent outpatient visit within a 4-day window. More than one diagnosis can be associated with an antibiotic prescription.

[c] Includes antibiotic prescriptions for any diagnosis.

[d] Includes diagnoses of pneumonia, sinusitis, pharyngitis, AOM, asthma/allergy, nonsuppurative otitis media, bronchitis/bronchiolitis, influenza, RSV, acute exacerbation of chronic bronchitis/obstructive pulmonary disorder (accounts for <0.04% of antibiotic prescriptions), and viral upper respiratory infection.

[e] Antibiotic prescriptions with concurrent diagnoses of urinary tract infection and other bacterial infections excluded.

[f] Includes diagnoses of bronchitis, bronchiolitis, and pneumonia excluding diagnosis codes for these conditions due to RSV or influenza.

[g] Excludes pneumonia due to influenza and RSV but includes other viral pneumonia. Among pneumonia prescriptions, 2.4% had a viral pneumonia diagnosis with no other pneumonia diagnosis.

[h] Excludes diagnosis codes for bronchiolitis due to RSV.

[i] Antibiotic prescriptions with concurrent diagnoses of urinary tract infection, other bacterial infections, pneumonia, AOM, sinusitis, pharyngitis, gastrointestinal infections, and skin and soft tissue infections excluded.

[j] Excludes diagnosis codes for bronchitis due to RSV.

[k] Includes all diagnoses of influenza, including influenza and pneumonia and influenza and AOM.

Table S7. Sensitivity analysis: model-estimated proportion of outpatient antibiotic prescriptions associated with influenza virus infection in analyses using bronchiolitis visits in children 0-2 years as respiratory syncytial virus incidence proxy.[a,b]

Age group	Percent of antibiotic prescriptions associated with infection (95% CI)
0-2 years	0.3 (-0.9, 1.4)
3-5 years	1.3 (0.2, 2.4)
6-9 years	3.4 (2.4, 4.4)
10-13 years	4.1 (3.4, 4.8)
14-17 years	3.3 (2.8, 3.7)
All children	3.1 (2.8, 3.5)

Abbreviations: CI – confidence interval

[a] Primary analysis used the percent of samples positive for RSV measured from laboratory data from NREVSS lagged one week behind antibiotic prescriptions as the RSV transmission proxy.

[b] Includes antibiotic prescriptions for any diagnosis.

Table S8. Incidence of respiratory syncytial virus-, influenza-, and bronchiolitis-associated visits by census division.

Census division	Visit incidence[a] per 1000 person-years (95% CI)[b]		
	RSV	*Influenza*	*Bronchiolitis*
East North Central	43 (31, 57)	80 (63, 98)	83 (66, 101)
East South Central	108 (89, 129)	219 (194, 248)	135 (113, 158)
Middle Atlantic	51 (38, 66)	97 (78, 117)	113 (93, 135)
Mountain	69 (54, 86)	89 (71, 108)	116 (96, 138)
New England	79 (62, 97)	97 (78, 117)	128 (107, 151)
Pacific	35 (24, 48)	63 (48, 79)	74 (58, 92)
South Atlantic	43 (31, 56)	124 (103, 147)	95 (76, 114)
West North Central	72 (56, 89)	105 (85, 125)	109 (90, 131)
West South Central	60 (46, 76)	191 (165, 219)	114 (94, 136)
Total	53 (39, 68)	121 (100, 143)	101 (82, 121)

Abbreviations: CI – confidence interval; RSV – respiratory syncytial virus.

[a] All visits with any diagnostic code for RSV, influenza, or bronchiolitis as detailed in **Table S3.** We additionally included visits with ICD-9-CM code 466.11 or ICD-10-CM code J21.0 (bronchiolitis due to RSV) in the definition of bronchiolitis visits.

[b] Obtained by fitting number of antibiotic prescriptions divided by person-years to a Poisson distribution, sampling 100,000 random draws, and obtaining the median, 2.5th and 97.5th percentile values.

Table S9. Model-estimated proportion of outpatient antibiotic prescriptions associated with respiratory syncytial virus and influenza virus infection, by census division.

Census division	Percent of antibiotic prescriptions associated with infection (95% CI)		Antibiotic prescription incidence per 1000 person-years (95% CI)	
	RSV	*Influenza*	*RSV*	*Influenza*
East North Central	3.4 (-1.0, 7.4)	3.2 (2.2, 4.3)	38.8 (-11.2, 84.3)	36.6 (24.8, 48.9)
East South Central	5.0 (1.1, 8.6)	4.1 (2.9, 5.3)	71.7 (16.0, 122.9)	59.2 (42.0, 76.9)
Middle Atlantic	6.7 (2.5, 10.6)	3.6 (2.5, 4.7)	76.2 (28.3, 120.7)	40.8 (27.9, 54.2)
Mountain	3.6 (-0.7, 7.5)	4.0 (2.8, 5.1)	36.6 (-6.7, 76.5)	40.4 (28.7, 52.6)
New England	5.2 (1.4, 8.6)	3.0 (2.1, 4.0)	52.8 (14.2, 88.4)	30.8 (21.2, 40.8)
Pacific	4.4 (0.2, 8.3)	3.5 (2.3, 4.7)	41.4 (2.2, 77.1)	32.9 (21.6, 44.6)
South Atlantic	7.3 (0.6, 13.4)	4.2 (2.9, 5.5)	85.1 (6.8, 157.0)	48.4 (33.2, 63.9)
West North Central	3.7 (-2.2, 9.0)	2.7 (1.7, 3.7)	43.9 (-26.7, 108.3)	31.7 (19.9, 44.0)
West South Central	5.3 (0.6, 9.6)	4.6 (3.3, 5.8)	71.5 (8.0, 129.8)	61.7 (44.6, 79.2)

Abbreviations: CI – confidence interval; RSV – respiratory syncytial virus.

Table S10. Sensitivity analysis – model-estimated proportion of outpatient antibiotic prescriptions associated with respiratory syncytial virus and influenza infection based on a linear model.

Age group	Associated fraction based on linear model,[a] % (95% CI)	
	RSV	*Influenza*
0-2 years	8.9 (7.1, 10.9)	1.3 (0.6, 2.0)
3-5 years	8.7 (6.9, 10.5)	1.9 (1.3, 2.5)
6-9 years	5.2 (3.3, 7.1)	3.5 (2.9, 4.1)
10-13 years	3.8 (2.2, 5.5)	4.2 (3.7, 4.6)
14-17 years	2.5 (1.5, 3.6)	3.5 (3.2, 3.8)
All children [b]	6.8 (6.0, 7.7)	3.1 (2.8, 3.3)

Abbreviations: CI – confidence interval; RSV – respiratory syncytial virus.

[a] Model of the incidence of antibiotic prescriptions (no. prescriptions/person-weeks) as a linear function of census division, 13-valent pneumococcal conjugate vaccine period, 1-week lagged percent of positive RSV tests, influenza incidence by age category, 12-, 6-, 4- and 3-month harmonics, and week secular trend term.

[b] The model for all children also controlled for age category.

Table S11: Sensitivity analysis – model-estimated proportion of outpatient antibiotic prescriptions associated with RSV and influenza infection, when antibiotic definition is expanded to all antibiotics.

**Age group**	**Fraction associated with each infection for all agents[a] % (95% CI)**	
	RSV	*Influenza*
0-2 years	8.4 (5.7, 11.1)	0.9 (-0.2, 2.0)
3-5 years	8.3 (5.5, 11.1)	1.7 (0.7, 2.8)
6-9 years	5.6 (3.0, 8.2)	3.6 (2.7, 4.5)
10-13 years	4.0 (2.3, 5.7)	4.1 (3.5, 4.6)
14-17 years	2.2 (1.0, 3.3)	3.0 (2.6, 3.4)
All children	6.1 (5.1, 7.1)	3.3 (3.0, 3.7)

Abbreviations: CI – confidence interval; RSV – respiratory syncytial virus.
[a] Includes all agents listed in **Table S2**.

Table S12. Sensitivity analysis — incidence rates and model-estimated proportion of outpatient antibiotic prescriptions associated with respiratory syncytial virus and influenza virus infection in analyses applying broader inclusion criteria for prescription plan utilization.

	**+/- 1-year Rx window***	**+/- 2-year Rx window***
N antibiotic prescriptions	18,466,530	18,466,530
N person-weeks	784,505,730	902,757,005
Approximate antibiotic prescription incidence per 1000 person-weeks	23.5	20.5
Proportion of antibiotic use associated with RSV (95% confidence interval)	6.3% (5.2-7.3%)	6.0% (4.7-7.2%)
Proportion of antibiotic use associated with influenza (95% confidence interval)	3.4% (3.1-3.8%)	3.7% (3.3-4.1%)

**Limited to study period of 2009-2017 to ensure capture of 2 years of prescription data for all cohort members.*

Table S13. Incidence and proportion of outpatient antibiotic prescriptions associated with respiratory syncytial virus and influenza infection linked to diagnoses for which antibiotics are not indicated, without exclusion of prescriptions linked to other conditions, among children aged 0-17 years, 2008-2018.

**Diagnosis**[a]	**Antibiotic prescriptions among children 0-17 years**		
	Incidence rate, per 1000 person-years (95% CI)[b]	*RSV-associated fraction, % (95% CI)*	*Influenza-associated fraction, % (95% CI)*
Bronchiolitis[c,d]	9 (4, 16)	24.2 (22.1, 26.1)	4.0 (3.1, 4.9)
Bronchitis[d,e]	61 (47, 77)	10.8 (8.9, 12.6)	6.0 (5.4, 6.7)
Nonsupp. OM[d]	48 (35, 63)	8.1 (6.8, 9.4)	2.2 (1.7, 2.7)
Allergy[d]	36 (25, 48)	4.1 (3.0, 5.2)	1.5 (1.1, 1.9)
Asthma[d]	38 (26, 50)	8.8 (7.4, 10.2)	3.5 (3.0, 3.9)
Viral URI (excl. RSV and influenza)[d]	199 (173, 228)	10.1 (8.6, 11.5)	6.7 (6.1, 7.2)
Any RSV diagnosis[d]	3 (0, 7)	49.8 (47.8, 51.7)	7.9 (6.6, 9.1)
Any influenza diagnosis[d,f]	9 (4, 15)	15.1 (13.2, 16.9)	92.0 (91.4, 92.6)

Abbreviations: RSV – respiratory syncytial virus; CI – confidence interval; Non-supp. OM – nonsuppurative otitis media; URI – upper respiratory infection.

[a] Based on ICD-9-CM and ICD-10-CM codes (**Table S3**) assigned at the most recent outpatient visit within a 4-day window. More than one diagnosis can be associated with an antibiotic prescription.

[b] Obtained by fitting number of antibiotic prescriptions divided by person-years to a Poisson distribution, sampling 100,000 random draws, and obtaining the median, 2.5th and 97.5th percentile values.

[c] Excludes diagnosis codes for bronchiolitis due to RSV.

[d] Antibiotic prescriptions with concurrent diagnoses of urinary tract and other bacterial infections excluded.

[e] Excludes diagnosis codes for bronchitis due to RSV.

[f] Includes all diagnoses of influenza, including influenza and pneumonia and influenza and AOM.

Figure S1. Predicted and observed antibiotic prescription counts

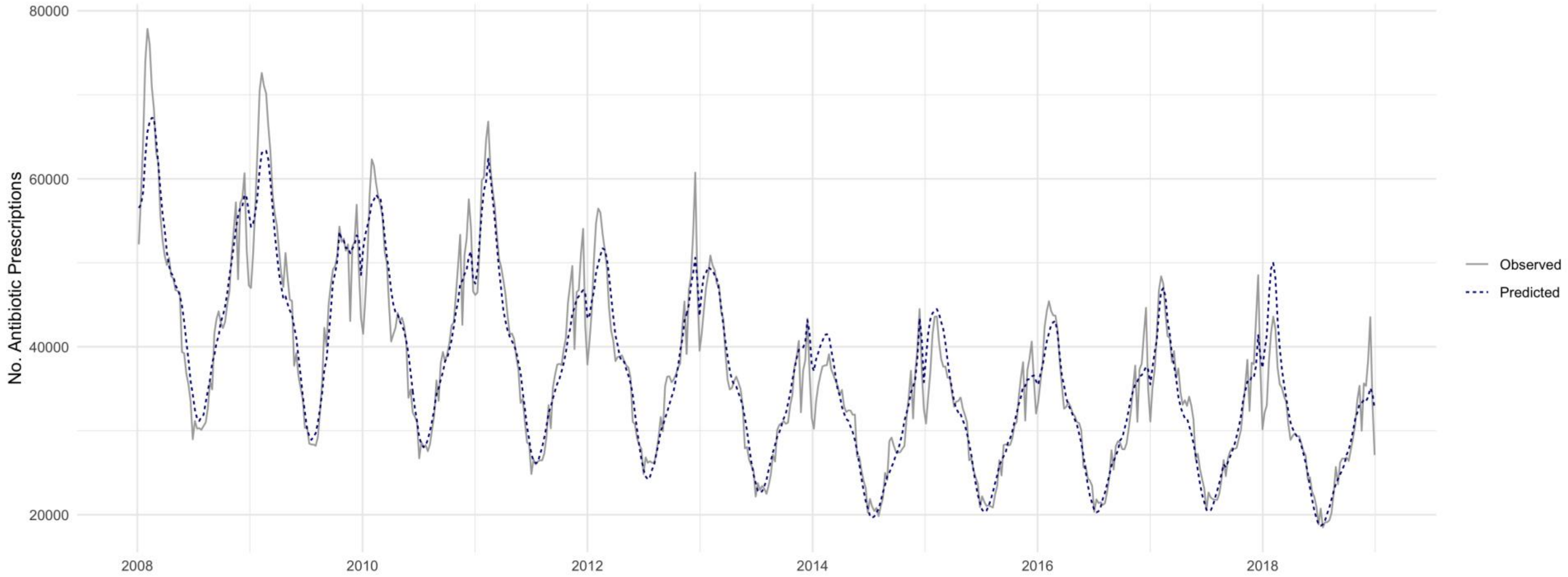

Supplement: jiaf309_Supplementary_Data [file jiaf309_supplementary_data.pdf]
